# Evidence at a glance: error matrix approach for overviewing available evidence

**DOI:** 10.1186/1471-2288-10-90

**Published:** 2010-10-01

**Authors:** Frederik Keus, Jørn Wetterslev, Christian Gluud, Cornelis JHM van Laarhoven

**Affiliations:** 1 The Cochrane Hepato-Biliary Group, Copenhagen Trial Unit, Centre for Clinical Intervention Research, Rigshospitalet, Copenhagen University Hospital, Copenhagen, Denmark; 2 The Department of Surgery of Radboud University Nijmegen Medical Center, Nijmegen, The Netherlands

## Abstract

**Background:**

Clinical evidence continues to expand and is increasingly difficult to overview. We aimed at conceptualizing a visual assessment tool, i.e., a matrix for overviewing studies and their data in order to assess the clinical evidence at a glance.

**Methods:**

A four-step matrix was constructed using the three dimensions of systematic error, random error, and design error. Matrix step I ranks the identified studies according to the dimensions of systematic errors and random errors. Matrix step II orders the studies according to the design errors. Matrix step III assesses the three dimensions of errors in studies. Matrix step IV assesses the size and direction of the intervention effect.

**Results:**

The application of this four-step matrix is illustrated with two examples: peri-operative beta-blockade initialized in relation to surgery versus placebo for major non-cardiac surgery, and antiarrhythmics for maintaining sinus rhythm after cardioversion of atrial fibrillation. When clinical evidence is deemed both internally and externally valid, the size of the intervention effect is to be assessed.

**Conclusion:**

The error matrix provides an overview of the validity of the available evidence at a glance, and may assist in deciding which interventions to use in clinical practice.

## Background

Evidence-based medicine (EBM) was first introduced in 1992 [1], and its increased application is reflected among others by the growth of The Cochrane Library databases as well as implementation of evidence-based guidelines into clinical practice [2]. EBM underpins that information provided from randomized trials, and systematic reviews of randomized trials represent the most reliable evidence regarding intervention effects [3,4]. Thanks to the sustained scientific process (Additional file 1: Table S1), we now know that the reliability of what we observe varies due to a whole array of different factors. There are three dimensions that particularly influence the reliability of our observations in clinical research and they are empirically and theoretically well accepted: the risk of systematic error ('bias'), the risk of random error ('play of chance'), and the risk of design error ('wrong design to answer the posed question') [4,9].

**Figure 1:**
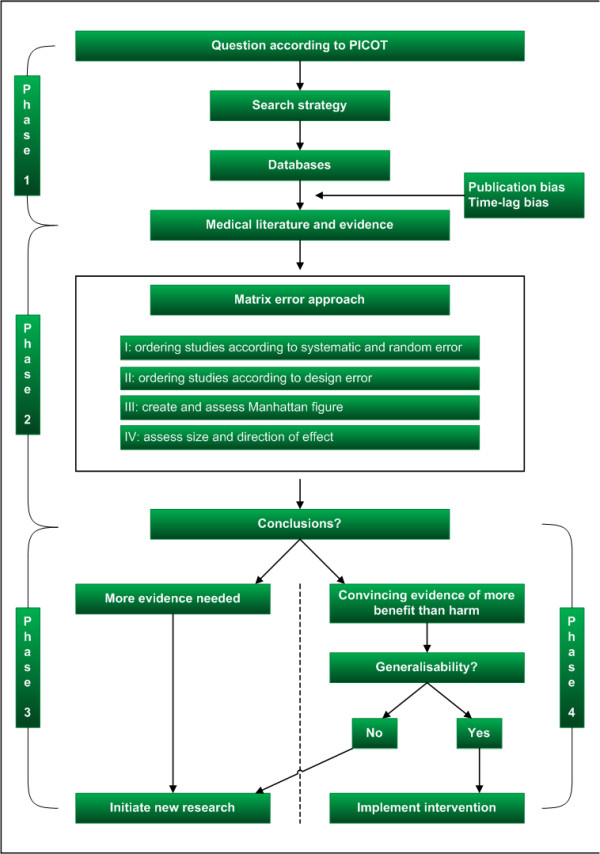
**Overview of the four phases in the process of evidence-based medicine from question to the initiation of new research or implementation of new evidence**. PICOT: patients, intervention, control, outcome measure, time.

EBM usually follows a four-phase process starting from a clinical question proceeding to the implementation of new evidence (Figure 1) [3]. Phase 1 is the formulation of a research question and literature search strategy. Phase 2 is the subsequent systematic appraisal and synthesis of the available evidence. Phase 3 covers the initiation of new research. Alternatively, phase 4 is the implementation of all available evidence when statistically and clinically convincing evidence has been obtained.

In daily clinical practice, the question of whether sufficient evidence is available to recommend the implementation of a specific intervention as a treatment arises frequently [3]. Depending on the specific clinical question, often an exhaustive list of references is retrieved when using a sensitive search strategy in multiple databases [3]. After the selection of studies, their data must be interpreted [10-13].

Since results may be contradictory and studies may differ in more than one aspect, to draw a clear, practical conclusion from the publications may be problematic [14].

### Objective

We aimed at conceptualizing a visual assessment tool, i.e., a matrix for overviewing studies and their data in order to assess the clinical evidence. The matrix is constructed from the three dimensions of errors: systematic error ('bias'), random error ('play of chance'), and design error ('wrong design to answer the question posed' or 'wrong context'). The application of this matrix will be illustrated by two examples: peri-operative beta-blockade initialized in relation to surgery versus placebo for major non-cardiac surgery, and antiarrhythmics for maintaining sinus rhythm after cardioversion of atrial fibrillation.

## Methods

### The three major error dimensions

#### The risk of systematic error ('bias')

When evaluating a clinical study, one should always try to assess its risk of systematic error [3,4,9-16]. There is increasing agreement on how trials and studies can be placed in a hierarchy when assessing the risk of systematic error [3,4,9-16], depending on the type of research (therapeutic, diagnostic, etiologic, or prognostic) [3,10,11,17]. The risk of systematic error influences the reliability of observed intervention effects [3,10,11,18,19]. A significant association between inadequate or unclear bias protection and overestimation of beneficial effects and underreporting of adverse effects has been demonstrated [16,19-23]. Differences in risk of bias are found both between the different levels of evidence and within each level of evidence [4,16,20].

For randomized trials, there is empirical evidence that at least six components are associated with systematic error: generation of the allocation sequence [24], allocation concealment [25], blinding [26], incomplete outcome measure reporting [4], selective outcome measure reporting [4], and other bias mechanisms (e.g., baseline imbalance, early stopping, vested interests, etc.) [4,16,20,27-29]. The impact of early stopping of trials on bias is largely dependent on how the stopping rules were defined and the level of statistical significance of the interim analysis [30-32]. Trials with one or more systematic error components assessed as inadequate or unclear are considered to be of high risk of bias, while trials with all quality components assessed as adequate are considered to be of low risk of bias [15,27,33]. Trials with a low risk of bias are more likely to estimate the 'true' effect of the intervention [16,20,27,33].

The systematic error dimension can be measured by an ordinal variable expressed in the levels of evidence (Table 1).

**Table 1:** Categorization of systematic error (bias) of clinical intervention studies into levels of evidence

Category	Studies
Level 1a	Meta-analysis of randomized trials with low risk of bias
Level 1b	Randomized trial with low risk of bias
Level 1c	Meta-analysis of all randomized trials
Level 1d	Randomized trial with high risk of bias
Level 2a	Meta-analysis of cohort studies
Level 2b	Cohort study
Level 3a	Meta-analysis of case-control studies
Level 3b	Case-control study
Level 4	Case-series
Level 5	Expert opinion

#### The risk of random error ('play of chance')

The risk of random error is the risk of drawing a false conclusion based on sparse data. There are two types of false conclusions: a false rejection of the null hypothesis (type I error; alpha) or a false acceptance of the null hypothesis (type II error; beta). When data are sparse, then the so called 'intervention effect', whether beneficial or harmful, may in fact be caused by randomly skewed variation in prognostic factors between the intervention groups due to sampling error.

The question, however, is how we quantify and compare the risk of random error between different studies with varying numbers of participants. A p-value reflects the risk that the difference in outcome between two interventions has arisen by chance, given the data and the null hypothesis are true. Since random low (and random high) p-values occur, especially during accumulation of data and sequential testing, the p-value does not sufficiently reflect the true risk of random error. Therefore, the p-values of intervention effect estimates certainly are not suitable for comparison of the risk of random error between different studies [32,34-37]. We suggest using the standard error (SE) for the evaluation of the risk of random error. We used the statistical algorithms from the statistical methods group of the Cochrane Collaboration [38]. The SE in a study may be considered a measure of uncertainty. The SE measures the amount of variability in the sample mean; it indicates how closely the population mean is likely to be estimated by the sample mean. The size of the standard error depends both on how much variation there is in the population and on the size of the sample. When two independent proportions *p*_*1 *_= *a/n*_*1 *_and *p*_*2 *_= *c/n*_*2 *_(with *a *and *c *being the numbers of patients with events, *b *and *d *being the numbers of patients with no events, and *n*_*1 *_and *n*_*2 *_being the total numbers of patients in the intervention group and control group, respectively) are considered in an individual study or a trial *i*, then the relative risk (*RR*_*i*_) is defined by:

$$RR_{i}=\frac{p_{1}}{p_{2}}$$

The SE of the log risk ratio for an individual study is calculated by the following formula:

$$SE\left[\ln\left(RR_{i}\right)\right]\;=\;\sqrt{\frac{1}{a_{i}}+\frac{1}{c_{i}}-\frac{1}{n_{1i}}-\frac{1}{n_{2i}}}$$

The Peto odds ratio (*OR*_*peto,i*_) for an individual study or trial *i *is defined by:

$$OR_{Peto,i}=\exp\left[\frac{Z_{i}}{V_{i}}\right]$$

where

$$Z_{i}=a_{i}-E\left[a_{i}\right]=a_{i}-\frac{n_{1i}(a_{i}+c_{i})}{N_{i}}\text{ and }V_{i}=\frac{n_{1i}\cdot n_{2i}\cdot (a_{i}+c_{i})\cdot (b_{i}+d_{i})}{N_{i}^{2}\cdot (N_{i}-1)}$$

The SE of the log Peto odds ratio for an individual study is defined by:

$$SE\left[Ln(OR_{Peto,i})\right]=\sqrt{\frac{1}{V_{i}}}$$

or

$$SE\left[Ln(OR_{Peto,i})\right]=\frac{N_{i}\sqrt{N_{i}-1}}{\sqrt{n_{1i}\cdot n_{2i}\cdot (a_{i}+c_{i})\cdot (b_{i}+d_{i})}}$$

In a meta-analysis results of studies or trials are meta-analysed into one intervention effect estimate. For the Mantel-Haenszel pooled risk ratio (*RR*_*MH*_) the natural logarithm of the *RR*_*MH *_has the standard error given by:

$$SE\left[\ln(RR_{MH})\right]=\;\sqrt{\frac{P}{R\cdot S}}$$

where

$$P=\sum_{i}\frac{n_{1i}\cdot n_{2i}\cdot \left(a_{i}+c_{i}\right)-a_{i}\cdot c_{i}\cdot N_{i}}{N_{i}^{2}}\text{ and }R=\sum_{i}\frac{a_{i}\cdot n_{2i}}{N_{i}}\text{ and }S=\sum_{i}\frac{c_{i}\cdot n_{1i}}{N_{i}}$$

and *N*_*i *_being the total number of patients in a trial.

For the pooled Peto OR (*OR*_*peto*_) the natural logarithm of the *OR*_*peto *_has the standard error given by:

$$SE\left[Ln(OR_{Peto})\right]=\sqrt{\frac{1}{\sum V_{i}}}$$

SE depends on the numbers of events and the sample size.

Due to spurious results, incorrect type I error inferences may be drawn. Recent reports indicate that the influence of the 'play of chance' may be much larger than generally perceived [39]. In randomized trials, random error may be one reason for the early stopping of trials at interim analyses when benefit or harm appear to be significant [32,40]. Increased random error may also play a role in the repeated analyses of accumulating data in both trials and meta-analyses [36,41-44]. A cumulative meta-analysis subjects accumulating data to repeated testing of the data and is bound to eventually lead to a false rejection of the null hypothesis ('false positive' result) [45,46]. The random error phenomenon or 'multiplicity' also plays a role in the evaluation of secondary outcome measures [40]. For example, when data on the primary research outcome, on which the sample size calculation was based, may not show statistical significance, while another outcome measure, for which no separate sample size calculation was performed, exhibits statistical significance [47,48].

Random error may be expressed in a continuous variable using the standard error of for example the log of Peto odds ratios or the log of relative risks.

#### The risk of design errors (external validity) - the participants included, the outcomes measured, the interventions, etc

When there is sufficient internal validity, i.e., low risks of systematic errors and random errors, it becomes relevant to consider the risks of design errors (external validity). The design (or context) of any piece of research determines its external validity or generalisability (Table 2) [4]. The external validity becomes questionable when a wrong design has been used to answer the question posed. Among the many variables that should be considered, the relevance of different outcome measures are of central importance to clinical research [13]. We, therefore, focus on them from a patient's perspective.

**Table 2:** Types of variables to consider when evaluating the risk of design errors ('context errors') and hence external validity of evidence

1	Outcome measures
2	Participants
3	Experimental intervention
4	Control intervention
5	Clinical centres or settings including patients
6	Goal - explanatory or pragmatic
7	Trial structure - parallel group, crossover, etc
8	Objective - superiority, equivalence, non-inferiority
9	Unit of analysis

**Figure 2:**
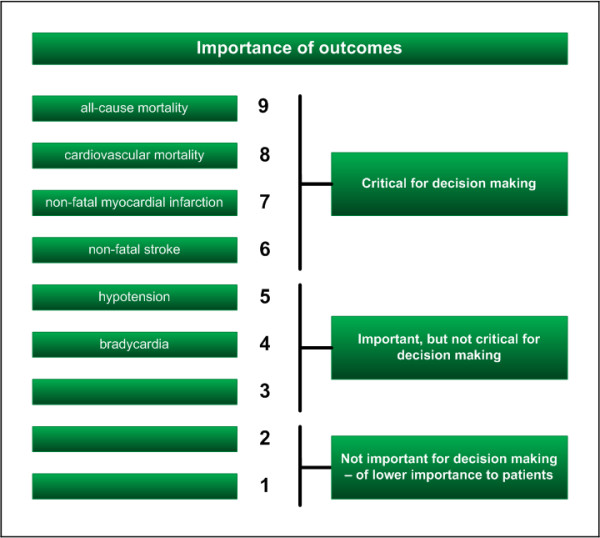
**Hierarchy of outcomes according to importance to non-cardiac surgery patients undergoing preventive beta-blocker intervention **[13]. Some outcome measures may be correlated (e.g. cardiovascular mortality is included in all-cause mortality)

Outcome measures can be divided into three categories according to the GRADE classifications (Figure 2) [13]. Primary outcome measures are central in deciding the use of one intervention over another. Large differences in the primary outcome measure between groups in a clinical trial may lead to early termination of a trial (following recommendations of a data safety and monitoring committee) [49]. Choice of the primary outcome should concur with the GRADE category of outcomes, 'critical for decision-making' [13]. Secondary outcome measures are additional outcome measures. If they are positively influenced by an intervention, the results may speak for recommending the intervention only if they support a beneficial effect on the primary outcome or if no clinically and statistically significant effect exist on the primary outcomes (e.g., a RR = 1.00 with 95% confidence limits from 0.98 to 1.02). The secondary outcomes should concur with the second and third GRADE categories of 'important, but not critical outcomes' [10-13].

GRADE has schematically ordered outcomes according to patients' perspective on a categorical scale from 1 to 9, with the most critical outcome, mortality, being graded 9 [13]. Depending on the outcomes, this scale should sometimes be considered nominal and in other situations be considered functional. Moreover, the severity of each outcome may differ as well. A stroke can be minor, while a myocardial infarction may involve a substantial worsening of cardiac function. Grading of outcome measures may also vary according to the clinical question. Therefore, outcomes within a category (i.e., critical, important, or not important) may be interchangeable. However, one can hardly argue that outcomes between categories (i.e., critical, important, or not important) are interchangeable (e.g., mortality is always more important than length of stay in hospital).

Eventually, the design error dimension can be expressed by the priority of outcome measures as an ordinal variable according to GRADE [13].

### Conceptualization of the error matrix

A four-step matrix can be constructed, building upon the three dimensions: systematic error, random error, and design error. Matrix step I ranks the identified studies according to the dimensions of systematic errors and random errors. Matrix step II orders the studies according to the design errors. Matrix step III assesses the three dimensions of errors in studies. Here, a 'Manhattan-like' error matrix is constructed where the best evidence is represented by the largest skyscrapers located on the 'upper-west side'. Matrix step IV assesses the size and direction of the intervention effect, e.g., by calculating the number-needed-to-treat to obtain benefit or to harm one patient.

The principle of the matrix approach can be used in different situations. The overall effort in research should be to minimize all three risks of errors before the size and the direction of the intervention effect can be assessed reliably. The 'algorithm' of the matrix approach is generally applicable to all kinds of interventions, although details may differ according to the specific clinical question. Or, the character of the three dimensions remains the same, while according to the specific question details may differ, like: the preferred hierarchy for levels of evidence, the chosen formula for standard error (*RR*, *OR*_*peto*_, or any other association metric), and the types of outcomes.

## Results

The application of this four-step matrix is illustrated with two examples: peri-operative beta-blockade initialized in relation to surgery versus placebo for major non-cardiac surgery, and antiarrhythmics for maintaining sinus rhythm after cardioversion of atrial fibrillation.

### Example 1: Initiating peri-operative beta-blockade for major non-cardiac surgery

A clinical question in PICOT structure illustrates this model. Is initiating peri-operative beta-blockade effective in patients undergoing major non-cardiac surgery?

**P**atients: patients undergoing major non-cardiac surgery; **I**ntervention: initiating peri-operative beta blockade; **C**ontrol: placebo; **O**utcome measure: mortality, myocardial infarction, and stroke; **T**ime: follow-up of at least 30 days.

We searched in CENTRAL in The Cochrane Library, PubMed, EMBASE, and personal files for all article types up to October 2009, in all languages. Specific searches using the terms 'beta-blockade', 'peri-operative', 'placebo', 'mortality', 'randomised', and 'non-cardiac surgery' were undertaken. The search resulted in multiple publications relevant to our question. References were selected from journals on the basis of importance and relevance [50-58]. We included the publications in our matrix evaluation by extracting information on all-cause mortality, cardiovascular mortality, non-fatal myocardial infarction, and non-fatal stroke. However, the matrix may easily be extended to other outcomes.

**Figure 3:**
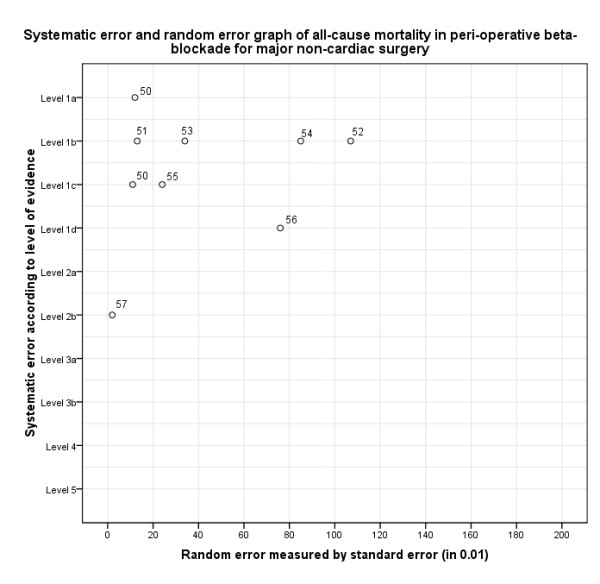
**Matrix step I, ordering of evidence according to systematic error (in levels of evidence) and random error (measured by standard error) considering all-cause mortality in peri-operative beta-blockade versus placebo for major non-cardiac surgery (example 1)**.

**Figure 4:**
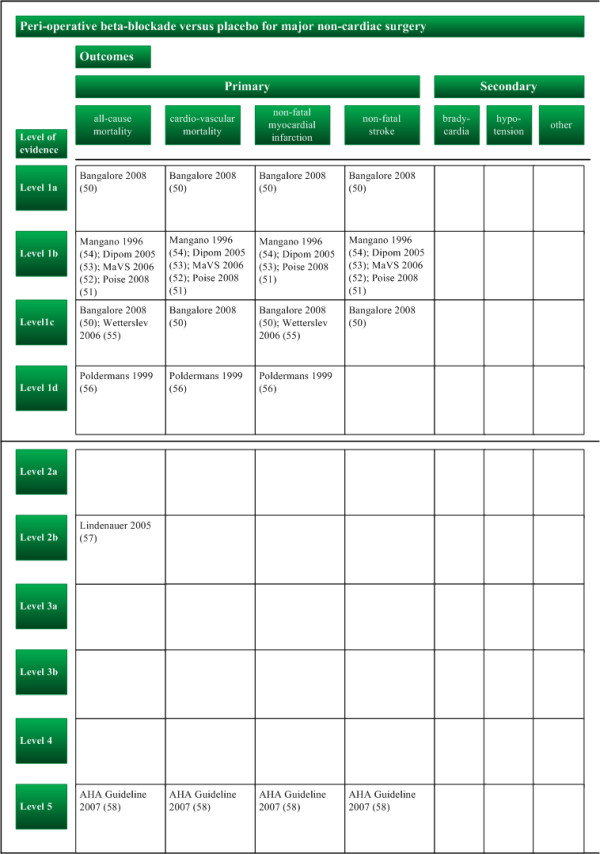
**Matrix step II, ordering of evidence on peri-operative beta-blockade versus placebo for major non-cardiac surgery according to importance of outcome measures (design error) and levels of evidence (systematic error) (example 1)**. The outcome measures have been adapted to the beta-blockade question.

**Figure 5:**
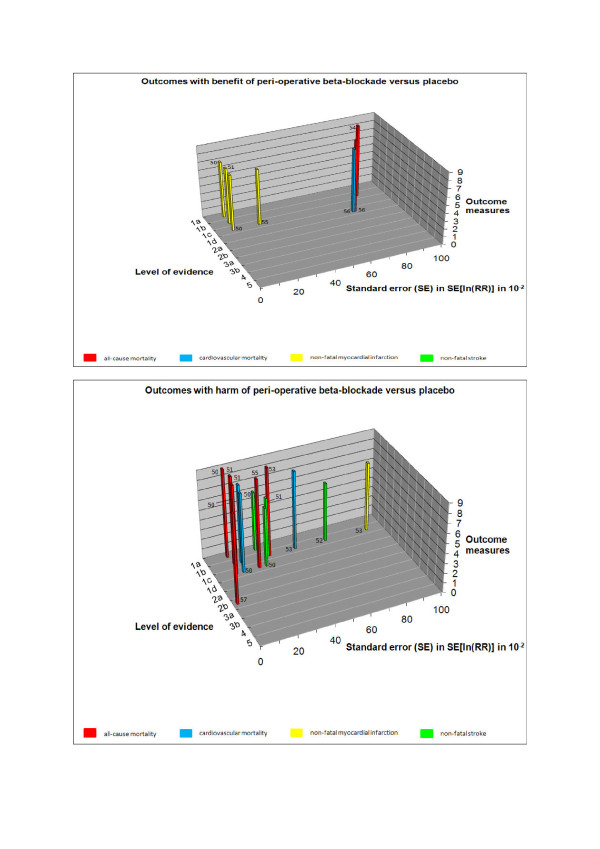
**Manhattan-like three-dimensional matrix building upon the risks of systematic error, random error, and design error.** The evidence with the lowest systematic, random, and design error is represented by the tallest skyscrapers, located on 'the upper west side'. **a**. Outcomes with benefit of peri-operative beta-blockade versus placebo. **b**. Outcomes with harm of peri-operative beta-blockade versus placebo. *A 'quick guide' to the perception of the figure: *If you want to know what the evidence is for peri-operative beta-blockade to influence myocardial infarction: go to the yellow bars and read 1) Level of evidence (the risk of systematic error) and 2) standard error (the risk of random error). Data with risk of systematic error >level 2b and random error SE >1.0 were omitted from the figure. The guidelines, which advocate the use of peri-operative beta-blockade, were not included in this figure since the systematic error is level 5 and the random error cannot be calculated (not based on data) [58]. The SE of outcomes with zero events cannot be calculated either. From these 'benefit' and 'harm' Manhattan figures, one can see at a glance that beta-blockers may provide benefit to patients in terms of nonfatal myocardial infarction (yellow bars). However, one can also see that beta-blockers may cause harm to patients in terms of all-cause mortality (red bars), cardiovascular mortality (blue bars), and nonfatal stroke (green bars). Reading the dimension of systematic error it is immediately clear that there is level 1a evidence available for all these four outcome measures. Reading the dimension of random error on this systematic error level of evidence shows that there is a small risk of random error considering all-cause mortality (0,12), cardiovascular mortality (0,16), and nonfatal myocardial infarction (0,10), and a moderate risk of random error considering nonfatal stroke (0,28). It is clear at a glance that the best available evidence does not support peri-operative beta-blockade for major non-cardiac surgery. SE = 0 to 0,10 = ignorable risk of random error. SE = 0,10 to 0,20 = small risk of random error. SE = 0,20 to 0,30 = moderate risk of random error. SE = 0,30 to 0,50 = substantial risk of random error. SE = >0,50 = high risk of random error. A clean version for creating a Manhattan figure can be obtained at the Copenhagen Trial Unit's homepage (http://ctu.rh.dk).

In step I, we assessed the systematic error and the random error for the chosen outcomes of each study (Figure 3, Table 3). In step II, we evaluated the design error (Figure 4). In step III, we constructed the three-dimensional matrix (Figure 5). We did not elaborate on the matrix step IV in this example.

**Table 3:** Ordering of evidence according to levels of evidence (systematic error), standard error (random error), and outcome measures (design error) in peri-operative beta-blockade versus placebo for major non-cardiac surgery (example 1).

	Level of evidence	Standard error			
		
		All-cause mortality	Cardiovascular mortality	Non-fatal myocardial infarction	Non-fatal stroke
Bangalore [50]	1a	0.12	0.16	0.10	0.28
Poise [51]	1b	0.13	0.17	0.10	0.33
MaVS [52]	1b	1.07	Z	N	0.66
Dipom [53]	1b	0.34	0.48	0.91	Z
Mangano [54]	1b	0.85	1.22	1.22	1.11
Bangalore [50]	1c	0.11	0.15	0.09	0.28
Wetterslev [55]	1c	0.24	N	0.23	N
Poldermans [56]	1d	0.76	0.76	Z	N
Lindenauer [57]	2b	0.02	N	N	N
AHA Guidelines [58]	5	N	N	N	N

Z: outcome with zero-events in one or both treatment arms which makes SE incalculable; N: no data

Some outcome measures may be correlated (e.g. cardiovascular mortality is included in all-cause mortality).

In this example the formulas for SE of lnRR_i _for individual studies and SE of lnRR_MH _for meta-analysis were used.

From Figure 5 it can be concluded at a glance that peri-operative beta blockade does not reduce mortality in patients undergoing major non-cardiac surgery. Peri-operative beta-blockade in these patients seems to increase all-cause mortality. However, peri-operative beta-blockade does reduce non-fatal myocardial infarction on the expense of an increased cardiovascular mortality and an increased rate of non-fatal stroke.

### Example 2: Antiarrhythmics for maintaining sinus rhythm after cardioversion of atrial fibrillation [59]

The conclusion of this Cochrane review focuses on the significant increased mortality associated with use of class 1a antiarrhythmics (odds ratio 2.39; 95% confidence interval (CI) 1.03 to 5.59) [59]. The data of this outcome in class 1a antiarrhythmics in this review [59] as well as in the included randomised trials [60-67] were analysed using the matrix error approach.

**Figure 6:**
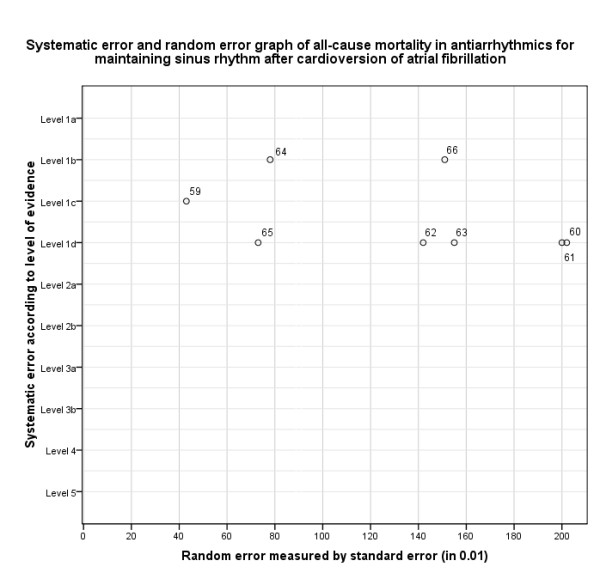
**Matrix step I, ordering of evidence according to systematic error (in levels of evidence) and random error (measured by standard error) considering all-cause mortality in antiarrhythmics for maintaining sinus rhythm after cardioversion of atrial fibrillation (example 2)**. Compare this figure with Figure 3: the studies in this figure are located on the right side of the figure (all SE >0.40), in contrast with Figure 3 where the studies are concentrated on the upper left side of the figure (six studies with SE < 0.40).>

**Figure 7:**
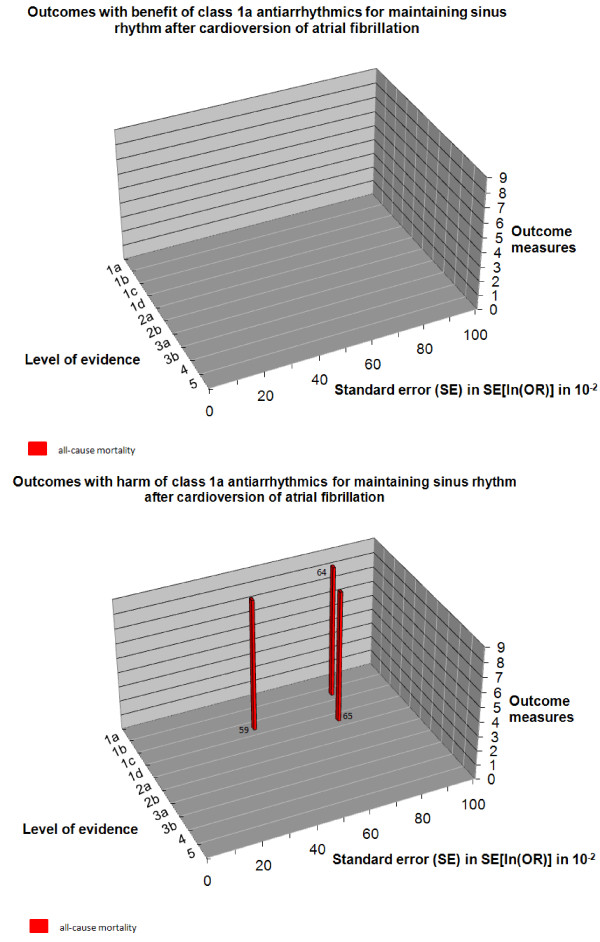
**Manhattan-like three-dimensional matrix building upon the risks of systematic error, random error, and design error.** The evidence with the lowest systematic, random, and design error is represented by the tallest skyscrapers, located on 'the upper west side'. **a**. Outcomes with benefit of antiarrhythmics for maintaining sinus rhythm after cardioversion of atrial fibrillation. **b**. Outcomes with harm of antiarrhythmics for maintaining sinus rhythm after cardioversion of atrial fibrillation. *A 'quick guide' to the perception of the figure: *If you want to know what the evidence is for antiarrhythmics for maintaining sinus rhythm after cardioversion of atrial fibrillation to influence all-cause mortality: go to the red bars and read 1) Level of evidence (the risk of systematic error) and 2) standard error (the risk of random error). Only the Cochrane review and the trials included in this systematic review were considered in this example. Data with risk of random error SE >1.0 were omitted from the figure. The SE of outcomes with zero events cannot be calculated. From these 'benefit' and 'harm' Manhattan figures, one can see at a glance that there is no benefit at all and that 'the upper west side' is empty. Class 1a antiarrhythmics might increase mortality; however, since high risks for both systematic error and random error are present there is insufficient evidence for reliable conclusions.

In step I, we assessed the risk of systematic error and the risk of random error for the chosen outcome of each study (Figure 6, Table 4). In step II, the design error should be evaluated by assessing multiple outcome measures. However, in this example we only consider the outcome 'all-cause mortality', since other outcomes were found to be not statistically significantly different [59]. Therefore, no figure of step II is shown. In step III, we constructed the three-dimensional matrix (Figure 7). We did not elaborate on the matrix step IV in this example, since the available studies are not internally valid (high risks of both systematic and random error).

**Table 4:** Ordering of evidence according to levels of evidence (systematic error), standard error (random error), and outcome measures (design error) in antiarrhythmics for maintaining sinus rhythm after cardioversion of atrial fibrillation (example 2)

	Level of evidence	Standard error
		**All-cause mortality**

Byrne-Quinn [60]	1d	2.02
Hillestad [61]	1d	2.00
Karlson [62]	1d	1.42
Lloyd [63]	1d	1.55
PAFAC [64]	1b	0.78
Sodermark [65]	1d	0.73
SOPAT [66]	1b	1.51
Steinbeck [67]	1d	Z
Lafuente-Lafuente [59]	1c	0.43

Z: outcome with zero-events in both treatment arms which makes SE incalculable

In this example the formulas for SE of lnOR_peto,i _for individual studies and SE of lnOR_peto _for meta-analysis were used.

From Figure 7 it can be concluded at a glance that there is both substantial risk of systematic and random error involved in the evidence available so far considering mortality associated with class 1a antiarrhythmics. The best available level of evidence 1c study shows substantial risk of random error (0.43) and the best available level of evidence 1b study shows high risk of random error (0.78). So, the conclusion in the Cochrane review of a significant increased mortality is based on data with high risks for both systematic and random errors, and should therefore be considered unreliable.

## Discussion

The aim of our matrix is to facilitate the overview of evidence in clinical intervention research. The matrix can serve as a tool to provide visual assessment of reliability of observations with respect to systematic error, random error (internal validity), and design error (external validity).

The matrix should not replace the thorough process of systematically reviewing evidence and profound evaluations of data, but could be integrated within these research activities as a tool for overviewing the results. Also, this matrix is not an absolute measure of the risks of errors. The position of studies in relation to each other is relative rather than absolute.

There is a lack of awareness of the importance of the 'play of chance' for the reliability of study findings. Ordering the standard errors of the studies might be a tool for ranking studies according to the level of random error. We have used natural logarithm (ln) transformations for calculating standard errors, although the logarithm with the base 10 may be used without producing different conclusions.

As an alternative, the Bayes factor can be considered [37,68]. The Bayes factor is a likelihood ratio comparing one hypothesis versus another, and, therefore, varies with the definition of the possible alternative hypotheses. The Bayes factor is a summary measure that provides an alternative to the p-value for the ranking or the flagging of associations as 'significant' [69]. The Bayes factor:

$$\text{Bayes factor}=\frac{\text{Probability }\left(\text{Data},\text{ given the null hypothesis}\right)}{\text{Probability }\left(\text{Data},\text{ given the alternative hypothesis}\right)}$$

or simple approximations can be very difficult or even impossible to implement for the clinician, since a search for the maximum of the multidimensional posterior may be required for each association [69]. This also includes the asymptotic Bayes factor introduced by Wakefield [69]. In contrast to the Bayes factor, it is possible to calculate the standard error and when available it provides a tool for comparison of the risk of random error between studies of the same intervention.

The aim of minimising error risks according to the three dimensions actually combines the methodological efforts of falsifying any alternative hypothesis in the evaluation of an intervention. Thereby, the matrix concept visualises how far the scientific process has evolved to fulfil Poppers falsification criterion stating that researchers should primarily engage trying to falsify any relevant alternative hypothesis and not only the null hypothesis [5]. The minimisation of systematic errors and random errors, by providing ample room for the null hypothesis, as well as measuring important outcomes is the most audacious attack on any realistic alternative hypothesis. If an array of progressively qualified attacks fails to support the null hypothesis then we can reliably trust the intervention to be either beneficial or harmful.

The conclusion based on an assessment of the evidence using the matrix approach may be implemented into clinical practice or serve as an incentive for new research. The matrix facilitates the identification of lacunae in our knowledge and is likely to benefit the process of developing evidence-based guidelines.

### Preference for the highest evidence

One has to be aware of the multiple forms of bias, potentially present in evidence below level 1 (Table 1). Several examples illustrate that large, apparently beneficial intervention effects from lower level evidence, even from randomized trials [54,56,70], may eventually be reversed to harmful effects when new high-quality evidence appears [50,71]. This is where the three dimensions of error are of central importance in providing a tool for reliability assessment.

### Limitations

Apart from the three error dimensions influencing the reliability of data, other factors play a role in incomparability and uncertainty of inferences. Many reports of studies appear incomplete, and the lack of details raises questions. Incomplete reporting limits interpretation, but more importantly, this reporting factor should be distinguished from the methodological quality of the trial [72].

Statements like CONSORT [73], PRISMA [74], and MOOSE [75] aim to improve and to maximize the amount and correctness of information to be retrieved from publications. These guidelines also create awareness among researchers about the most important issues to report so that the quality of future research may increase. By following reporting guidelines the yield of the research question is likely to be increased (phase 1 in Figure 1).

Standard error does not consider testing of multiple outcomes and multiple testing on accumulating data, which may also induce risks of random error due to multiplicity as well as correlations.

The division of all outcomes into 'primary' and 'secondary' outcome measures can be helpful as this division sets the standards for the evaluation of interventions. However, this division is artificial, and outcome measures, situated on the border of primary and secondary outcomes, exist. For example, one can argue that quality of life is a primary outcome rather than a secondary outcome. Further, there is also a quantitative aspect in the artificial division into primary and secondary outcomes. Small significant differences in primary outcome measures (e.g., bile duct injuries in patients undergoing cholecystectomy) may be found favouring one intervention, while large differences in secondary outcome measures (e.g., costs) may favour the comparator. Eventually, one may prefer the larger advantages in secondary outcomes to the smaller disadvantages in a primary outcome measure.

Another limitation in the outcome measure dimension is that often outcome measures are correlated and mostly this correlation is ignored. For example when mortality is an outcome measure and complications is another, which again counts deaths as complications, then there is a correlation between the two outcome measures. Authors usually carry out multiple univariate analyses ignoring correlations between outcome measures.

Step IV of the matrix includes the assessment of the size of the intervention effect, e.g., expressed in numbers-needed-to-treat to obtain benefit or to harm one patient with the intervention. This step is the last one since it is irrelevant to consider effect sizes and their directions if a study does not appear to be internally and externally valid.

Another aspect to consider is heterogeneity [76,77]. Statistical heterogeneity reflects the between trial variance of meta-analytic intervention effect estimates rather than the play of chance [76]. Clinical heterogeneity, however, represents differences in populations, procedures, or interventions in daily practice. All these factors of clinical heterogeneity, together with concordance of in- and exclusion criteria should be considered whenever we want to implement results of available evidence. Assessment and consideration of heterogeneity or diversity, therefore, forms the final step before new evidence is implemented. Assessment of heterogeneity is not included in our matrix.

## Conclusions

Assessment of risks of systematic error, random error, and design error are essential factors in evaluating evidence and drawing conclusions. We used the standard error in our matrix to rank studies according to their risk of random error. The risks of these error types were incorporated into a three dimensional matrix to create a schematic overview of the internal and external validity of the evidence, seen at a glance.

## Competing interests

The authors declare that they have no competing interests.

## Authors' contributions

FK, JW, CG, and CL contributed to the development of the ideas in the manuscript. FK and JW performed the analyses, created the figures, and drafted the original text. CG and CL commented and contributed to the discussion. All authors read and approved the final manuscript.

## Funding

There was no funding.

## Pre-publication history

The pre-publication history for this paper can be accessed here:

http://www.biomedcentral.com/1471-2288/10/90/prepub

## Supplementary Material

Additional file 1 **Table S1**. Word DOC table showing the sustained scientific process. Click here for file

## References

[B1] Evidence-Based Medicine Working Group Evidence-based medicine. A new approach to teaching the practice of medicine JAMA 1992 268 2420 5 10.1001/jama.268.17.2420 1404801

[B2] Sutton AJ Higgins JPT Recent developments in meta-analysis Stat Med 2008 27 625 50 10.1002/sim.2934 17590884

[B3] Straus SE Richardson WS Glasziou P Haynes RB Evidence-based medicine. How to practice and teach EBM 2005 Edinburgh, UK: Churchill Livingstone

[B4] Higgins JPT Green S Cochrane Handbook for Systematic Reviews of Interventions 2008 The Cochrane Collaboration

[B5] Popper KR Logik der Forschung 1959 Vienna: Springer

[B6] Kuhn T The Structure of Scientific Revolutions 1962 Chicago: The University of Chicago Press

[B7] Sehon SR Stanley DE A philosophical analysis of the evidence-based medicine debate BMC Health Serv Res 2003 3 14 10.1186/1472-6963-3-14 12873351 PMC169187

[B8] Quine WV From a logical point of view 1953 Cambridge: Harvard University Press

[B9] Gluud C The culture of designing hepato-biliary randomised trials J Hepatol 2006 44 607 15 10.1016/j.jhep.2005.12.006 16434120

[B10] Atkins D Eccles M Flottorp S Guyatt GH Henry D Hill S Liberati A O'Connell D Oxman AD Phillips B Schünemann H Edejer TT Vist GE Williams JW Jr GRADE Working Group Systems for grading the quality of evidence and the strength of recommendations I: critical appraisal of existing approaches. The GRADE Working Group BMC Health Serv Res 2004 4 38 10.1186/1472-6963-4-38 15615589 PMC545647

[B11] Atkins D Briss PA Eccles M Flottorp S Guyatt GH Harbour RT Hill S Jaeschke R Liberati A Magrini N Mason J O'Connell D Oxman AD Phillips B Schünemann H Edejer TT Vist GE Williams JW Jr GRADE Working Group Systems for grading the quality of evidence and the strength of recommendations II: pilot study of a new system BMC Health Serv Res 2005 5 25 10.1186/1472-6963-5-25 15788089 PMC1084246

[B12] Guyatt GH Oxman AD Vist GE Kunz R Falck-Ytter Y Alonso-Coello P Schünemann HJ GRADE Working Group GRADE: an emerging consensus on rating quality of evidence and strength of recommendations BMJ 2008 336 924 6 10.1136/bmj.39489.470347.AD 18436948 PMC2335261

[B13] Guyatt GH Oxman AD Kunz R Vist GE Falck-Ytter Y Schünemann HJ GRADE Working Group What is "quality of evidence" and why is it important to clinicians? BMJ 2008 336 995 8 10.1136/bmj.39490.551019.BE 18456631 PMC2364804

[B14] Grimes DA Schulz KF An overview of clinical research: the lay of the land Lancet 2002 359 57 61 10.1016/S0140-6736(02)07283-5 11809203

[B15] Kjaergard LL Villumsen J Gluud C Reported methodologic quality and discrepancies between large and small randomized trials in meta-analyses Ann Intern Med 2001 135 982 9 11730399 10.7326/0003-4819-135-11-200112040-00010

[B16] Schulz KF Chalmers I Hayes RJ Altman DG Empirical evidence of bias. Dimensions of methodological quality associated with estimates of treatment effects in controlled trials JAMA 1995 273 408 12 10.1001/jama.273.5.408 7823387

[B17] Glasziou P Vandenbroucke JP Chalmers I Assessing the quality of research BMJ 2004 328 39 41 10.1136/bmj.328.7430.39 14703546 PMC313908

[B18] Altman DG Randomisation. Essential for reducing bias BMJ 1991 302 1481 2 10.1136/bmj.302.6791.1481 1855013 PMC1670173

[B19] Kunz R Vist G Oxman AD Randomisation to protect against selection bias in healthcare trials Cochrane Database of Methodology Reviews 2002 4 10.1002/14651858.MR000012.pub2 17443633

[B20] Wood L Egger M Gluud LL Schulz KF Jüni P Altman DG Gluud C Martin RM Wood AJ Sterne JA Empirical evidence of bias in treatment effect estimates in controlled trials with different interventions and outcomes: meta-epidemiological study BMJ 2008 336 601 5 10.1136/bmj.39465.451748.AD 18316340 PMC2267990

[B21] Grimes DA Schulz KF Bias and causal associations in observational research Lancet 2002 359 248 52 10.1016/S0140-6736(02)07451-2 11812579

[B22] Grimes DA Schulz KF Cohort studies: marching towards outcomes Lancet 2002 359 341 5 10.1016/S0140-6736(02)07500-1 11830217

[B23] Grimes DA Schulz KF Descriptive studies: what they can and cannot do Lancet 2002 359 145 9 10.1016/S0140-6736(02)07373-7 11809274

[B24] Schulz KF Grimes DA Generation of allocation sequences in randomised trials: chance, not choice Lancet 2002 359 515 9 10.1016/S0140-6736(02)07683-3 11853818

[B25] Schulz KF Grimes DA Allocation concealment in randomised trials: defending against deciphering Lancet 2002 359 614 8 10.1016/S0140-6736(02)07750-4 11867132

[B26] Schulz KF Grimes DA Blinding in randomised trials: hiding who got what Lancet 2002 359 696 700 10.1016/S0140-6736(02)07816-9 11879884

[B27] Moher D Jadad AR Tugwell P Assessing the quality of randomized controlled trials. Current issues and future directions Int J Technol Assess Health Care 1996 12 195 208 10.1017/S0266462300009570 8707495

[B28] Jadad AR Moore RA Carroll D Jenkinson C Reynolds DJ Gavaghan DJ McQuay HJ Assessing the quality of reports of randomized clinical trials: is blinding necessary? Control Clin Trials 1996 17 1 12 10.1016/0197-2456(95)00134-4 8721797

[B29] Gluud LL Bias in clinical intervention research Am J Epidemiol 2006 163 493 501 10.1093/aje/kwj069 16443796

[B30] Mueller PS Montori VM Bassler D Koenig BA Guyatt GH Ethical issues in stopping randomized trials early because of apparent benefit Ann Intern Med 2007 146 878 81 17577007 10.7326/0003-4819-146-12-200706190-00009

[B31] Goodman SN Stopping at nothing? Some dilemmas of data monitoring in clinical trials Ann Intern Med 2007 146 882 7 17577008 10.7326/0003-4819-146-12-200706190-00010

[B32] Montori VM Devereaux PJ Adhikari NK Burns KE Eggert CH Briel M Lacchetti C Leung TW Darling E Bryant DM Bucher HC Schünemann HJ Meade MO Cook DJ Erwin PJ Sood A Sood R Lo B Thompson CA Zhou Q Mills E Guyatt GH Randomized trials stopped early for benefit: a systematic review JAMA 2005 294 2203 9 10.1001/jama.294.17.2203 16264162

[B33] Jüni P Altman DG Egger M Systematic reviews in health care: Assessing the quality of controlled clinical trials BMJ 2001 323 42 6 10.1136/bmj.323.7303.42 11440947 PMC1120670

[B34] Bassler D Ferreira-Gonzalez I Briel M Cook DJ Devereaux PJ Heels-Ansdell D Kirpalani H Meade MO Montori VM Rozenberg A Schünemann HJ Guyatt GH Systematic reviewers neglect bias that results from trials stopped early for benefit J Clin Epidemiol 2007 60 869 73 10.1016/j.jclinepi.2006.12.006 17689802

[B35] Bassler D Montori VM Briel M Glasziou P Guyatt GH Early stopping of randomized clinical trials for overt efficacy is problematic J Clin Epidemiol 2008 61 241 6 10.1016/j.jclinepi.2007.07.016 18226746

[B36] Wetterslev J Thorlund K Brok J Gluud C Trial sequential analysis may establish when firm evidence is reached in cumulative meta-analysis J Clin Epidemiol 2008 61 64 75 10.1016/j.jclinepi.2007.03.013 18083463

[B37] Goodman SN Toward evidence-based medical statistics. 1: The P value fallacy Ann Intern Med 1999 130 995 1004 10383371 10.7326/0003-4819-130-12-199906150-00008

[B38] Deeks J Higgins J on behalf of the statistical methods group of the Cochrane collaboration Standard statistical algorithms in Cochrane reviews, Version 5 2005 The Cochrane Collaboration http://ims.cochrane.org/revman/documentation/Statistical-methods-in-RevMan-5.pdf

[B39] Vickers AJ Underpowering in randomized trials reporting a sample size calculation J Clin Epidemiol 2003 56 717 20 10.1016/S0895-4356(03)00141-0 12954462

[B40] Schulz KF Grimes DA Multiplicity in randomised trials II: subgroup and interim analyses Lancet 2005 365 1657 61 10.1016/S0140-6736(05)66516-6 15885299

[B41] Pogue JM Yusuf S Cumulating evidence from randomized trials: utilizing sequential monitoring boundaries for cumulative meta-analysis Control Clin Trials 1997 18 580 93 10.1016/S0197-2456(97)00051-2 9408720

[B42] Brok J Thorlund K Gluud C Wetterslev J Trial sequential analysis reveals insufficient information size and potentially false positive results in many meta-analyses J Clin Epidemiol 2008 61 763 9 10.1016/j.jclinepi.2007.10.007 18411040

[B43] Thorlund K Devereaux PJ Wetterslev J Guyatt GH Ioannidis JP Thabane L Gluud LL Als-Nielsen B Gluud C Can trial sequential monitoring boundaries reduce spurious inferences from meta-analyses? Int J Epidemiol 2009 38 276 86 10.1093/ije/dyn179 18824467

[B44] Brok J Thorlund K Wetterslev J Gluud C Apparently conclusive meta-analyses may be inconclusive - Trial sequential analysis adjustment of random error risk due to repetetive testing of accumulating data in apparently conclusive neonatal meta-analyses Int J Epidemiol 2009 38 287 98 10.1093/ije/dyn188 18824466

[B45] Berkey CS Mosteller F Lau J Antman EM Uncertainty of the time of first significance in random effects cumulative meta-analysis Control Clin Trials 1996 17 357 71 10.1016/S0197-2456(96)00014-1 8932970

[B46] Lau J Schmid CH Chalmers TC Cumulative meta-analysis of clinical trials builds evidence for exemplary medical care J Clin Epidemiol 1995 48 45 57 10.1016/0895-4356(94)00106-Z 7853047

[B47] O'Neill RT Secondary endpoints cannot be validly analyzed if the primary endpoint does not demonstrate clear statistical significance Control Clin Trials 1997 18 550 6 10.1016/S0197-2456(97)00075-5 9408717

[B48] Schulz KF Grimes DA Multiplicity in randomised trials I: endpoints and treatments Lancet 2005 365 1591 5 10.1016/S0140-6736(05)66461-6 15866314

[B49] Friedman LM Furberg CD Demets DL Fundamentals of clinical trials 1998 New York: Springer Verlag

[B50] Bangalore S Wetterslev J Pranesh S Sawhney S Gluud C Messerli FH Peri-operative beta-blockers in patients undergoing non-cardiac surgery. A meta-analysis and trial sequential analysis of 12,306 patients from randomised trials Lancet 2008 372 1962 76 10.1016/S0140-6736(08)61560-3 19012955

[B51] POISE Study Group Devereaux PJ Yang H Yusuf S Guyatt G Leslie K Villar JC Xavier D Chrolavicius S Greenspan L Pogue J Pais P Liu L Xu S Málaga G Avezum A Chan M Montori VM Jacka M Choi P Effects of extended-release metoprolol succinate in patients undergoing non-cardiac surgery (POISE trial): a randomised controlled trial Lancet 2008 371 1839 47 10.1016/S0140-6736(08)60601-7 18479744

[B52] Yang H Raymer K Butler R Parlow J Roberts R The effects of perioperative beta-blockade: results of the Metoprolol after Vascular Surgery (MaVS) study, a randomized controlled trial Am Heart J 2006 152 983 90 10.1016/j.ahj.2006.07.024 17070177

[B53] Juul AB Wetterslev J Gluud C Kofoed-Enevoldsen A Jensen G Callesen T Nørgaard P Fruergaard K Bestle M Vedelsdal R Miran A Jacobsen J Roed J Mortensen MB Jørgensen L Jørgensen J Rovsing ML Petersen PL Pott F Haas M Albret R Nielsen LL Johansson G Stjernholm P Mølgaard Y Foss NB Elkjaer J Dehlie B Boysen K Zaric D DIPOM Trial Group Effect of perioperative beta blockade in patients with diabetes undergoing major non-cardiac surgery: randomised placebo controlled, blinded multicentre trial BMJ 2006 332 1482 10.1136/bmj.332.7556.1482 16793810 PMC1482337

[B54] Mangano DT Layug EL Wallace A Tateo I Effect of atenolol on mortality and cardiovascular morbidity after noncardiac surgery. Multicenter Study of Perioperative Ischemia Research Group N Engl J Med 1996 335 1713 20 10.1056/NEJM199612053352301 8929262

[B55] Wetterslev J Juul AB Benefit and harms of perioperative b-blockade Best Pract Res Clin Anaesthesiol 2006 20 285 302 10.1016/j.bpa.2005.10.006 16850778

[B56] Poldermans D Boersma E Bax JJ Thomson IR van de Ven LL Blankensteijn JD Baars HF Yo TI Trocino G Vigna C Roelandt JR van Urk H The effect of bisoprolol on perioperative mortality and myocardial infarction in high-risk patients undergoing vascular surgery. Dutch Echocardiographic Cardiac Risk Evaluation Applying Stress Echocardiography Study Group N Engl J Med 1999 341 1789 94 10.1056/NEJM199912093412402 10588963

[B57] Lindenauer PK Pekow P Wang K Mamidi DK Gutierrez B Benjamin EM Perioperative beta-blocker therapy and mortality after major noncardiac surgery N Engl J Med 2005 353 349 61 10.1056/NEJMoa041895 16049209

[B58] Fleisher LA Beckman JA Brown KA Calkins H Chaikof EL Fleischmann KE Freeman WK Froehlich JB Kasper EK Kersten JR Riegel B Robb JF Smith SC Jr Jacobs AK Adams CD Anderson JL Antman EM Buller CE Creager MA Ettinger SM Faxon DP Fuster V Halperin JL Hiratzka LF Hunt SA Lytle BW Nishimura R Ornato JP Page RL Riegel B ACC/AHA 2007 Guidelines on Perioperative Cardiovascular Evaluation and Care for Noncardiac Surgery: Executive Summary: A Report of the American College of Cardiology/American Heart Association Task Force on Practice Guidelines (Writing Committee to Revise the 2002 Guidelines on Perioperative Cardiovascular Evaluation for Noncardiac Surgery) Developed in Collaboration With the American Society of Echocardiography, American Society of Nuclear Cardiology, Heart Rhythm Society, Society of Cardiovascular Anesthesiologists, Society for Cardiovascular Angiography and Interventions, Society for Vascular Medicine and Biology, and Society for Vascular Surgery J Am Coll Cardiol 2007 50 1707 32 10.1016/j.jacc.2007.09.001 17950159

[B59] Lafuente-Lafuente C Mouly S Longas-Tejero MA Bergmann JF Antiarrhythmics for maintaining sinus rhythm after cardioversion of atrial fibrillation Cochrane Database Syst Rev 2007 4 CD005049 17943835 10.1002/14651858.CD005049.pub2

[B60] Byrne-Quinn E Wing AJ Maintenance of sinus rhythm after DC reversion of atrial fibrilllation. A double-blind controlled trial of long-acting quinidine bisulphate Br Heart J 1970 32 370 6 10.1136/hrt.32.3.370 4911757 PMC487336

[B61] Hillestad L Bjerkelund C Dale J Maltau J Storstein O Quinidine in maintenance of sinus rhythm after electroconversion of chronic atrial fibrillation. A controlled clinical study Br Heart J 1971 33 518 21 10.1136/hrt.33.4.518 4934041 PMC487206

[B62] Karlson BW Torstensson I Abjorn C Jansson SO Peterson LE Disopyramide in the maintenance of sinus rhythm after electroconversion of atrial fibrillation. A placebo-controlled one year follow-up study Eur Heart J 1988 9 284 90 3289932 10.1093/oxfordjournals.eurheartj.a062498

[B63] Lloyd EA Gersh BJ Forman R The efficacy of quinidine and disopyramide in the maintenance of sinus rhythm after electroconversion from atrial fibrillation. A double-blind study comparing quinidine, disopyramide and placebo S Afr Med J 1984 65 367 9 6367096

[B64] Fetsch T Bauer P Engberding R Koch HP Lukl J Meinertz T Oeff M Seipel L Trappe HJ Treese N Breithardt G Prevention of Atrial Fibrillation after Cardioversion Investigators Prevention of atrial fibrillation after cardioversion: results of the PAFAC trial Eur Heart J 2004 25 1385 94 10.1016/j.ehj.2004.04.015 15302102

[B65] Södermark T Jonsson B Olsson A Orö L Wallin H Edhag O Sjögren A Danielsson M Rosenhamer G Effect of quinidine on maintaining sinus rhythm after conversion of atrial fibrillation or flutter. A multicentre study from Stockholm Br Heart J 1975 37 486 92 10.1136/hrt.37.5.486 1093559 PMC482826

[B66] Patten M Maas R Bauer P Lüderitz B Sonntag F Dluzniewski M Hatala R Opolski G Müller HW Meinertz T SOPAT Investigators Suppression of paroxysmal atrial tachyarrhythmias-results of the SOPAT trial Eur Heart J 2004 25 1395 404 10.1016/j.ehj.2004.06.014 15321697

[B67] Steinbeck G Doliwa R Bach P Therapy of paroxysmal atrial fibrillation. Cardiac glycosides alone or combined with antiarrhythmia agents? Dtsch Med Wochenschr 1988 113 1867 71 10.1055/s-2008-1067903 3143539

[B68] Goodman SN Toward evidence-based medical statistics. 2: The Bayes factor Ann Intern Med 1999 130 1005 13 10383350 10.7326/0003-4819-130-12-199906150-00019

[B69] Wakefield J Bayes factors for genome-wide association studies: comparison with P-values Genet Epidemiol 2008 33 79 86 10.1002/gepi.20359 18642345

[B70] Deeks JJ Dinnes J D'Amico R Sowden AJ Sakarovitch C Song F Petticrew M Altman DG International Stroke Trial Collaborative Group; European Carotid Surgery Trial Collaborative Group Evaluating non-randomised intervention studies Health Technol Assess 2003 7 iii-x,1-173. 14499048 10.3310/hta7270

[B71] Jüni P Nartey L Reichenbach S Sterchi R Dieppe PA Egger M Risk of cardiovascular events and rofecoxib: cumulative meta-analysis Lancet 2004 364 2021 9 10.1016/S0140-6736(04)17514-4 15582059

[B72] Devereaux PJ Choi PT El Dika S Bhandari M Montori VM Schünemann HJ Garg AX Busse JW Heels-Ansdell D Ghali WA Manns BJ Guyatt GH An observational study found that authors of randomized controlled trials frequently use concealment of randomization and blinding, despite the failure to report these methods J Clin Epidemiol 2004 57 1232 6 10.1016/j.jclinepi.2004.03.017 15617948

[B73] Schulz KF Altman DG Moher D CONSORT Group CONSORT 2010 Statement: updated guidelines for reporting parallel group randomised trials BMC Med 2010 8 18 http://www.consort-statement.org 10.1186/1741-7015-8-18 20619135

[B74] Moher D Liberati A Tetzlaff J Altman DG Group PRISMA Preferred reporting items for systematic reviews and meta-analyses: the PRISMA statement BMJ 2009 339 b2535 http://www.prisma-statement.org 10.1136/bmj.b2535 19622551 PMC2714657

[B75] Stroup DF Berlin JA Morton SC Olkin I Williamson GD Rennie D Moher D Becker BJ Sipe TA Thacker SB Meta-analysis of observational studies in epidemiology: a proposal for reporting JAMA 2000 283 2008 12 10.1001/jama.283.15.2008 10789670

[B76] Higgins JP Thompson SG Quantifying heterogeneity in a meta-analysis Stat Med 2002 21 1539 58 10.1002/sim.1186 12111919

[B77] Wetterslev J Thorlund K Brok J Gluud C Estimating required information size by quantifying diversity in random-effects model meta-analyses BMC Med Res Methodol 2009 9 86 10.1186/1471-2288-9-86 20042080 PMC2809074

