# Supplementary material for: Evidence at a glance: error matrix approach for overviewing available evidence
Source: BMC Med Res Methodol. 2010 Oct 1;10:90. doi: 10.1186/1471-2288-10-90 (PMC2959031; doi:10.1186/1471-2288-10-90)
Supplement: Additional file 1 — Table S1. Word DOC table showing the sustained scientific process. [file 1471-2288-10-90-S1.DOC]

***Table S1.***

| Many consider evidence-based medicine (EBM) a paradigm shift in medicine. However, EBM should rather be considered a continuation of the way of thinking that evolved from rationalistic optimist philosophers like Thomas Kuhn and Karl Popper [5,6]. They introduced modern concepts of paradigm shift and critical rationalism and stressed the importance of scientific experiments or trials in order to challenge ‘normal science’ and gain reliable knowledge. EBM is part of this development, which is underpinned by the teaching that clinical knowledge based on randomized trials and systematic reviews of randomized trials represent the most reliable evidence [3,4]. In accordance, Sehon and Stanley [7] state that EBM should not be considered a Kuhnian paradigm shift [6], but should more readily be seen in the light of the ‘Quinean doctrine of holism’ [8]. The ‘web of belief’ metaphor of Quine integrates all fundamental different approaches and underlines the dependency of all alternative approaches on each other [7,8]. EBM rather provides categorization of results based on more or less controlled observations fitting in the ‘web of belief’. Analogous to this, our presented matrix could be considered a helpful tool in categorizing evidence and providing graphical visualization. Although we recognize the value of alternative approaches in medicine, we consider that the solid ground, which EBM provides, is the approach with which to go forward. We strongly recommend against ‘evidence shopping’ for the evidence one may eventually find to support a prejudiced view, ignoring the possible lack of evidence at a higher level (that is jumping the fence where it is lowest). This seems reasonable both when best available evidence is present, but also when best obtainable evidence is within reach with some extra reasonable efforts. |
| --- |
